# Why New Hybrid Organizations are Formed: Historical Perspectives on Epistemic and Academic Drift

**DOI:** 10.1007/s11024-013-9226-x

**Published:** 2013-04-16

**Authors:** Thomas Kaiserfeld

**Affiliations:** Department of Arts and Cultural Sciences, Lund University, Biskopsgatan 7, 223 62 Lund, Sweden

**Keywords:** Hybrid organizations, Epistemic drift, Academic drift, Scientific academies, Vocational training, Industrial research institutes

## Abstract

By comparing three types of hybrid organizations—18th-century scientific academies, 19th-century institutions of higher vocational education, and 20th-century industrial research institutes—it is the purpose here to answer the question of why new hybrid organizations are continuously formed. Traditionally, and often implicitly, it is often assumed that emerging groups of potential knowledge users have their own organizational preferences and demands influencing the setup of new hybrid organizations. By applying the concepts epistemic and academic drift, it will be argued here, however, that internal organizational dynamics are just as important as changing historical conjunctures in the uses of science when understanding why new hybrid organizations are formed. Only seldom have older hybrid organizations sought to make themselves relevant to new categories of knowledge users as the original ones have been marginalized. Instead, they have tended to accede to ideals supported by traditional academic organizations with higher status in terms of knowledge management, primarily universities. Through this process, demand has been generated for the founding of new hybrid organizations rather than the transformation of existing ones. Although this study focuses on Swedish cases, it is argued that since Sweden strove consistently to implement existing international policy trends during the periods in question, the observations may be generalized to apply to other national and transnational contexts.

## Introduction

The call for users of research to step forward and make their voices heard, both to counter the threat of a technocracy run loose and to keep the otherworldly tendencies of scientists in check, was not a new phenomenon in the 1990s or even the 19th century. In fact, users have always been an important feature of scientific culture (Hessels and van Lente 2008; Smith 1994; Porter 1995; Brown 2009a). Despite the focus on new roles for science in society, academic disciplines, and even epistemology in the literature on the boundaries of science, this article will show that a discourse revolving around the idea of the user as central to scientific endeavours, either as a rhetorical device or constructed for specific purposes, has been essential for centuries in creating organizations concerned with both the use and generation of new scientific knowledge (Hellström and Jacob 2003).

In general, these types of historical organizations can be equated with hybrid organizations, that is, organizations relying on a combination of social practices drawn from the worlds of both science and politics (Miller 2001).^1^ A basic observation is that since older hybrid organizations prevail as new ones are introduced, they form historical layers like superposed sediments.^2^ By analyzing how these bodies have been created historically, stretching back beyond the Cold War era and even the 19th century, it is the purpose of this article to help to explain why new hybrid organizations have continuously been formed in ever-changing shapes and contexts since at least early modernity.

### *Epistemic and Academic Drift*

In order to do this, a preliminary discussion regarding two concepts is necessary. The first of these, *epistemic drift*, will be applied to denote a process by which the criteria scientists use to assess the value of research problems and results, rather than being established entirely through internal protocols such as peer-review, are transformed so that scientists tend to place greater weight on the relevance of their research for politically, administratively, or commercially determined goals.^3^ As has often been observed, the analytical distinction between external and internal relevance is difficult, if not impossible, to draw and maintain (Shapin 1992). Nevertheless, analyses of the symbolic function of the potential users and uses of research can be exploited in order to characterize the notion of epistemic drift without having to work out the boundaries between external and internal epistemic criteria.^4^ From such a perspective, epistemic drift can be defined as processes by which values from ideological systems external to science, for instance business or policy, are adopted by researchers making them pay increasing attention to the potential uses of their activities and practices.

Originally, the concept of epistemic drift was developed to describe a state-driven process of increasing political influence over research agendas in Sweden in the 1970s and 1980s (Elzinga 2010). But using the somewhat broader definition given here, epistemic drift can be applied more extensively to denote any process where interests other than those of scientists influence scientific research, its results and the assessment of those results. These user-induced interests have, of course, transformed over time and thus given rise to demands for new hybrid organizations. But as will be shown here, this only partially accounts for the formation of new hybrid organizations. In addition to conjunctures in the uses of science, many hybrid organizations themselves follow a path that seems to make room for new ones to appear.

In this article, epistemic drift is thus used to denote the process in which researchers and the representatives of other interests interact in order to generate new knowledge and make it more accessible to potential users. More specifically, the focus here is on how these processes may lead to the formation of new hybrid organizations. Of course, the balance between different stakeholders participating in processes of epistemic drift varies from one historical context to another. These stakeholders may also have varying agendas, whether hidden or explicit. As has been pointed out many times, the notions of the *exclusivity* and the *usefulness* of knowledge about natural phenomena both have long traditions stretching back to the late Renaissance or earlier (Hannaway 1986; Dear 2005). These two ideal types of ideological underpinnings of science, framed in concepts such as the *vita contemplativa* and the *vita activa*, have to varying degrees been the ideals of both the producers and the users of knowledge over the past centuries. The concept of epistemic drift is used here to describe a process leading to an increased focus on potential uses. In this way, it points out the direction of a process, but reveals nothing about its starting point.

The concept of epistemic drift is balanced by a second concept, *academic drift*, conventionally defined as a process entailing an increased valuation and assimilation of academic practices.^5^ Traditionally, academic drift has been used to describe and analyze tendencies within vocational education, typically engineering schools of different levels (Harwood 2006). The problem, then, is to pinpoint the meaning and content of academic practice, not an easy task considering the various meanings the term *academic* has been given throughout modern history. It is clear, however, that academic drift is used to describe a situation where institutions for vocational training pursue research and teaching based to a large extent on intellectual education and book learning rather than practice, irrespective of whatever else is implied by the term *academic* in a given context. Here, science is seen as a superior way of solving problems, while more practice-oriented actors view science as only one tool among others (Harwood 2005). Thus the concept of academic drift is also a rhetorical instrument connecting certain criteria for assessing the value of education to academic traditions. Keeping this in mind, academic drift can be defined as a process by which the practitioners of science pay increasing attention to scholarly procedures and routines, including the search for knowledge for knowledge’s sake, while paying less attention to the potential uses of these activities and practices. Note that the scope of academic drift can easily be expanded to include other organizations. As we will see, many different types of hybrid organizations can be viewed as exposed to academic drift, at least when followed over appropriately extended periods of time.

By bringing these two concepts together, it is possible to analyze processes that seem to counteract each other. On the one hand, academic drift can be viewed as the result of more or less successful endeavours to normalize an ideal of secluded research, where experimentation rather than experience is the primary mode of observation in labs isolated from the buzz and chatter of the outside world, and where the communication of interpretations and results in research networks for standardized data collection is judged more important than demands for external relevance (Callon, Lascoumes, and Barthe 2009: 37–70). Of course, the outside world is always there inside the lab, not only as noise and disturbance, but also as an inevitable influence and directing force. Still, academic drift is the result of more or less successful *endeavours* to normalize an *ideal* of seclusion. Epistemic drift, on the other hand, is the result of more or less successful *endeavours* to normalize an *ideal* of relevance to the outside world. Here, the mutual engagement of different interests in research networks is seen as a seal of legitimacy, and the results presented are assessed accordingly. Clearly, the concepts of epistemic and academic drift as defined here refer to two opposing and thus mutually exclusive processes, and it could be argued that the use of one of these concepts alongside the observation that the process it denotes is reversible would suffice. Here, both concepts will be used nevertheless, primarily for the sake of clarity, but also to show how they can be used in tandem. Note though, that while mutually exclusive, these two concepts are not jointly exhaustive. There are other forms of organizational drift that can occur that are neither epistemic nor academic. For instance, academic practices at universities can drift towards ideals stressing pedagogical skills at the expense of subject didactics or transdisciplinarity at the expense of traditional disciplines, both transforming academic values, but in other ways than epistemic drift would imply.

### *Long-Term Transformations of Hybrid Organizations*

The central observation underpinning the argument of this article—indeed, the observation which makes it possible to compare hybrid organizations over extended periods of time, even over the course of centuries—is that outwardly differing historical forms of hybrid organizations such as scientific academies, institutions for higher vocational education, and industrial research institutes have one important feature more or less in common. They have all been founded in general as organizational solutions to a specific perceived problem: that of a great divide disconnecting, on the one side, the researchers who seek seclusion in order to obtain knowledge potentially valuable to users in different areas of economic life, and, on the other side, the users who are believed to be in no position to influence the focus of this research in order to suit their own purposes. In short, scientific academies, institutions for higher vocational education, and industrial research institutes have all more often than not been set up as hybrid organizations in order to bridge a perceived divide between the producers and potential users of knowledge.

When studying the circulation of scientific truth-values and trust-values over the span of centuries, continuous epistemic authority (in the traditional meaning of the ability to determinatively influence the formation and use of knowledge) often turns out to be an important condition for hybrid organizations to last for those extended time periods needed in order to drift, either academically or epistemically (Pierson 1994). In its turn, epistemic authority is closely related to perceived reliability, relevance, and social robustness, all of which are needed for successful knowledge transfer over boundaries separating the producers from the users of knowledge and thus for the success of organizational and institutional hybridity (Nowotny 2003; Nowotny, Scott, and Gibbons 2001). In each historical context, however, epistemic authority has been understood differently. In addition, it has been related in different ways to the authority of other societal sectors, such as military power and coercion, religious beliefs, or professional organizations (Brown 2009b). Nevertheless, in all its diverse shapes and forms, epistemic authority is essential for making organizations successful in the transference of knowledge. In an environment of changing relations between different types of authorities, this means that hybrid organizations have to be dynamic for the preservation of epistemic authority while simultaneously maintaining social robustness.

Sharing these prerequisites, the long-term transformations of hybrid organizations are seldom random, but seem to follow trends best described by the concept of academic drift. This does not imply that academic drift is inevitable for hybrid organizations. On the contrary, as Jonathan Harwood (2010) has argued for the case of higher professional education in the fields of agriculture, engineering, medicine, and management, certain features serve to strengthen the tendency for academic drift, features he has used to explain why academic drift has occurred in some institutions and not others. According to Harwood, there are different strategies used by hybrid organizations when seeking epistemic authority. One is to seek recognition within academic hierarchies, either informal and unofficial or state-sanctioned, as was often the case in European settings. Another is to seek recognition within the realm of potential groups of users such as professional organizations. Yet another strategy is to seek access to material resources. In Harwood’s analysis, an organization’s position in the hierarchy of academic status, its geographic location, and the financial and political implications that these entail, determine to a large extent its tendency to drift in one direction or the other. More specifically, organizations only loosely connected to activities in the surrounding region tend to drift academically, as do organizations that have a high or moderate academic status. Conversely, organizations that are well-connected regionally and have a low academic status tend to drift epistemically. Especially interesting in this context is that Harwood’s model can be generalized to encompass other types of hybrid organizations.

Bruce Seely (1993) describes another dimension of this dynamic in his study of academic drift in American engineering colleges, where he notes an escalating interest in scientific inquiry and a marked increase in theoretical subjects in course curricula over the first half of the 20th century. By stretching the time frame forward in a later study, he has been able to show that the stress of American engineering education oscillated between theory and practice over the course of the century. According to Seely (1999), engineering education was practice-oriented in the early 20th century, but became more focused on research during the mid-century through the influence of European-educated engineers with a more analytical and mathematical approach to the subject. Later, however, the pendulum swung back and there was a renewed interest in more practice-oriented education among American engineers. Seely explains this phenomenon as a result of differences between European and American engineers by pointing out that Americans, from simple technicians to those holding a doctorate, were all educated in the same institutions, while a more heterogeneous educational system for engineers was in place in Europe where theoretical perspectives had higher status. When European influence over American engineering education peaked in mid-century, the result was thus a leaning towards the theoretical parts of curricula.

Taken together, it is clear from the findings of Harwood and Seely that academic drift is not inevitable. Instead, there are clear indications that these processes are driven by an organization’s position in a status hierarchy, its geographic location, and its inclination to conform to prevailing perceptions of the relation between practice and theory. It is therefore important to point out that all the hybrid organizations dealt with here are easily recognized as having had strong tendencies towards academic drift according to Harwood’s model of institutional dynamics. They all had prominent positions in the national hierarchy of academic status and were all located in the national capital. There were, of course, other less renowned hybrid organizations as well. These were less inclined towards academic drift, in accordance with Harwood’s model, due to their weak positions in the prevailing status hierarchy. This lack of status corresponded to a lack of epistemic authority to survive as functioning hybrid organizations over extended periods of time. It is equally clear that the engineering colleges used as examples here were part of a heterogeneous system of educational institutions, as Seely argues was the case in Europe in general (Torstendahl 1975). It would therefore be wrong to claim that all hybrid organizations drift academically. They certainly do not. It would, however, accord with the results of Harwood and Seely to claim that hybrid organizations with the necessary epistemic authority and social robustness to survive for centuries tend to drift academically.

In the following historical analysis of the creation and drift of a few hybrid organizations stretching from the 18th to the 20th century, the focus will be on Swedish examples, but the conclusions drawn have strong general implications. The reason is that Sweden has throughout its modern history implemented pre-existing policy trends with astounding consistency, making the country a model of the Western world in general (Elzinga 1984; Kaiserfeld 2010). And in terms of national research policies, the country has often served as a mirror for measures taken previously elsewhere. In this, Sweden is not very different from many other small Western countries where problems and solutions in science policy seem to appear almost simultaneously during the past centuries, a phenomenon often referred to as policy convergence (Wittrock 1984; Lemola 2002). By examining how and why hybrid organizations were founded in Sweden from the 18th century onwards, as well as by delineating how they drifted after their founding, it is the purpose here to propose an answer to the more general problem of why new generations of hybrid organizations continuously supplant one another, an answer that will point to organizational dynamics rather than changing historical conjunctures in the uses of science. The focus of this analysis will primarily be on 18th-century scientific academies, 19th-century institutions for higher vocational education, and 20th-century industrial research institutes.

Although this article deals with historical hybrid organizations, there are a number of different presently active hybrid organizations engaged in bridging a perceived knowledge producer-user division. One example is supplied by research councils often used to launch politically initiated programmes to distribute funding to politically selected research problems with more or less explicit demands for the participation of user categories (Jacob 2005). One other important institution for hybridity is constituted by the different types of forums for lay people interaction with researchers and politicians materialized in open hearings, citizen panels etc. (Maasen and Weingart 2005; Callon, Lascoumes, and Barthe 2009). Best known are perhaps different patient organization movements slowly transforming the relations between medical research, the medicine industry, medical doctors and their patients (Landzelius 2006). Whether these and other more recent organizations will drift, and if so which way, is, however, a matter for future analysis since patterns of epistemic or academic drift are only discernable through long-term historical analysis.

## Scientific Academies of the 18th Century

According to the chronicler of 18th-century scientific academies, James E. McClellan III (1985), approximately seventy such academies were established between 1660 and 1793. Modelled after the Royal Society of London and the French Academy of Sciences, they formed a collective unity sharing common members and undertaking common projects. Among their common features were legal charters granted by some civil authority (such as a king), systems of self-government set down in written rules, officers and elected fellow members who met regularly, and activities such as prize competitions and published transactions or memoirs. These official academies were complemented by private organizations of a similar character.

When surveying the landscape of scientific academies and societies of the 18th century, McClellan noted that academies were more common in countries where absolute monarchies ruled and agriculture dominated economic life, while societies modelled on the royal in London more often were oriented towards industry, trade and the sea. The academies of Berlin and of the Swedish capital Stockholm were, however, pointed out as more ambiguous from this perspective (p. 13). Nevertheless, when describing the hierarchy of status prevailing between the different scientific academies and societies of the 18th century, he placed the Royal Swedish Academy of Sciences (Kungliga Vetenskapsakademien) at the top together with those of London, Paris, Berlin, and St. Petersburg (McClellan III 1985: 34).

Although the founding of the Royal Swedish Academy of Sciences in 1739 was influenced by all of these international precedents, not least in picking up the thread of Baconian empiricism that tied them together, it proved initially to be very different in its even more marked emphasis on utilitarian and economic goals (Henry 1999). Swedish precursors, the Uppsala-based Collegium Curiosorum formed in 1710 and the Societas Scientiarum founded in the 1720s, had functioned in the same vein, albeit on a more restricted scale (Hildebrand 1939a, b: 88-94; Liljencrantz 1939; Liljencrantz 1940). And there would prove to be derivatives as well, most notably the Royal Society of Arts and Sciences (Kungliga Vetenskaps- och Vitterhets-Samhället) formed in 1778 in the commercial port city of Gothenburg (Eriksson 1978). Thus the Royal Swedish Academy of Sciences appeared neither in an international nor a national vacuum. Moreover, the histories of these contemporary societies and academies reveal similar developments over time.

### *The Royal Swedish Academy of Sciences as a Hybrid Organization*

The name *Vetenskapsakademien* can be interpreted as ‘knowledge society’, given that *vetenskap* (roughly, ‘science’; compare the German *Wissenschaft*) had a broader range of meaning than it does today, and given that the word *akademi* resonated more with the French notion of *société* than the 18th-century Swedish meaning of a university or school in general (von Höpken 1739). The name nevertheless continued to connote an alternative type of university concerned with the discovery and propagation of new and useful knowledge, in contrast to the traditional notion of the university in which knowledge was disseminated primarily through the teaching of established curricula. In fact, the Academy was originally proposed to be named the ‘Economic Scientific Society’ (Oeconomisk Wetenskaps Societet) in accord with the ideological predilections of many of its founding fathers, most notably Carl Linnaeus, who claimed that science was primarily an instrument for economic mercantilism and patriotism.^6^ The first paragraph of its rules stated that only the arts and sciences ‘possessing real utility for the commonwealth’ were to be the subject of the Academy’s attention.^7^

Already from the start, the Academy published transactions with original articles in Swedish intended to be read in wider circles, a strategy well in line with the principle of utility (Liedman 1989). Space was certainly reserved in these transactions for more specialized pieces on topics ranging over the whole fields of natural history and philosophy, but popular texts aimed at a wider audience—often dealing with agricultural topics—dominated throughout the 18th century (Bergström 2000). Thus, the transactions of the academy were indisputably saturated with research problems and results evaluated on the basis of their relevance to politically and administratively determined goals making them exponents of epistemic drift. The suggestions to translate work from the publications of the Royal Society and the French Academy were, however, never realized (Hildebrand 1939a, b: 230-31).

During its first years the Academy was heterogeneous in its makeup, one historian characterizing it as ‘a mixed congregation’.^8^ Counts and cabinet ministers collaborated with tax collectors; professors renowned on the Continent exchanged thoughts with apothecaries and accountants. The mix mirrored scientific practice since learned discourse could seldom be clearly distinguished from the world of commerce and politics (Klein and Spary 2009). Highlighting its heterogeneous character, a contemporary witness described its membership as comprising both ‘protectors and protected’, with representatives of political and commercial life on one side and natural philosophers on the other.^9^

This amalgam of scientific and economic interests was upheld through an election process in which a new candidate nominated by an elected member had to be approved by at least a three-fourths majority of those present (Lindroth I 1967: 12-5). Two and a half years after the Academy’s foundation in 1739, its membership had already grown to 64 and five years later it had increased to 94 (Lindroth I 1967: 27-30). By 1818 the number of elected members reached 383, nearly a fifth of whom were aristocrats, landed gentry, high-ranking state officials, and military commanders. Almost as large was the group of university professors and teachers. Other occupational groups represented were low-ranking public officials, physicians, and artisans. Throughout this period, the ratio between the different categories remained more or less constant (Lindroth II 1967: 28-9, 75-80, 91).

### *The Academic Drift of the Academy*

The drift of the Academy began in the 1820s when its heterogeneous character started to slowly dissolve. During the 1820s, 30s, and 40s, university teachers constituted approximately one-third of the new domestic recruits. In the following decade, this percentage increased considerably, so that two-thirds or more of new members were active at Swedish universities (Dahlgren 1915; Lindroth II 1967: 573-5). Changes could also be detected in the published transactions, where articles aimed at a wider audience became less frequent at the expense of those written for specialist readers. One reason was a shortage of articles during the early 19th century, which compelled the editors to refashion the transactions into a more attractive publication for scholars who otherwise preferred to publish their results in international journals (Lindroth II 1967: 76, 123-6). While the Royal Swedish Academy of Sciences started off as a very clear case of a hybrid organization, it certainly became less so during the 19th century due to academic drift, here measured primarily by the changing composition of its body of members and the articles published in its transactions.

It is hard to reconstruct the historical factors behind the academic drift of the Royal Swedish Academy of Sciences. One feature to take into account is the election system by which new candidates were nominated and elected by existing ones. Assuming that members had a tendency to vote for newcomers with a background resembling their own, this system was at an unstable equilibrium as long as the different member categories were reasonably proportionate to each other. Once one category started to grow, however, it could outnumber the others in a relatively short period of time. The jump from one-third of new domestic recruits of the Academy being university teachers in the 1840s to two-thirds in the 1850s could be understood as a result of the expansion of the Swedish system of higher education in the mid-19th century, especially in the Stockholm-Uppsala area, supplying a growing stock of university teachers to choose members from, and a subsequent shift in the proportion of university teachers making the numbers of this category tip over in their favour (Blomqvist 1992). It is important to point out that although these explanations partly rely on local non-generalizable factors, the expansion of different national systems of higher education and especially the natural sciences, occurred in many different countries in the mid-19th century as did the academic drift of scientific academies founded in the 16th and 17th centuries.

Already by the 1810s and 20s, however, academy members had started to import into Sweden a new type of hybrid organization they had observed in Berlin. As a result, engineering schools were organized in Stockholm to instruct artisans and managers connected to ‘industries grounded on chemical and physical foundations’.^10^ This was not the Academy’s first attempt to create spin-off hybrid organizations. It had earlier taken over botanical gardens and initiated libraries, 18th-century institutions in which knowledge was managed partly with users in mind.^11^ Moreover, the Academy’s attempt to import organized engineering training was part of a larger European trend in the 19th century to establish new educational institutions aimed at spreading what was thought to be useful knowledge. Higher vocational education was in vogue all over the Western world, and Sweden was no exception. Notably, however, the interest in vocational training in areas such as agriculture, medicine, and technology coincided with the Academy’s exposure to academic drift.

## 19th-Century Institutions of Higher Vocational Education

During the first half of the 19th century, the interest in engineering education went hand in hand with the gradual introduction of chemical and mechanical industries, as well as new methods of transportation such as the steam engine. In fact, as the golden era of Swedish natural history and philosophy began to decline in the 1780s or even earlier, technical and industrial endeavours seem to have become of greater ideological importance (Johannisson 1979-1980; Lindqvist 1989: 121). And as the Academy slowly lost its character as a hybrid organization, institutions for engineering training seem to have been more reasonable candidates for making use of knowledge.

In Sweden, as in many other European countries, a number of institutions for vocational education were formed during the 19th century. This process has been analyzed from a wide range of perspectives, having been depicted, for example, as a response to the demands of industrialized society, or as a form of rationalized education developed under the auspices of an expanding state (Day 1987; Artz 1966). The ideological and social underpinnings of educational efforts were of course relevant since they determined which vocations were deemed worthy of receiving higher educational institutions. But the existence of ideological and social platforms did not guarantee that the resulting organizations remained true to the original arguments and ideals concerning educational practices. In fact, in almost all the differing historical instances of vocational training, a central struggle can be discerned between the proponents of theoretical knowledge generated through scientific methods and their opponents, the practice-oriented seekers of know-how (cf. Gispen 1989; Grattan-Guinness 2005). In their recurring struggles over curricular content, institutions for vocational education have served as good examples of hybrid organizations in which practices have been drawn from the worlds of both science and politics.

### *Higher Vocational Education in Sweden as Hybrid Organizations*

The role and use of knowledge were thus central topics in the discussions preceding the establishment of vocational training institutions in different contexts, for example, the Stockholm University College of Physical Education and Sports (Gymnastiska centralinstitutet) in 1813, the Caroline Medical Institute (Kungliga Karolinska medico-kirurgiska institutet) in 1819-22, the Technological Institute (Teknologiska institutet) in 1827, the Chalmers Institute (Chalmerska institutet) in 1829, the Forestry Institute (Skogsinstitutet) in 1828, and the Pharmaceutical Institute (Farmaceutiska institutet) in 1837 (Anon 1913; Johannisson, Nilsson and Qvarsell 2010; Lagerkvist 1999; Henriques 1917; Bodman 1929; Lagerberg 1928; Fries and Zimmerman 1978; Ekström and Danielsson 1987). This list, far from complete, gives only a hint of the 19th-century interest in forming new establishments of this kind, many of which were originally intended for education on lower levels as well. In general, the teachers of these institutions were titled professors and their background was, with some variation, from the universities as PhDs or as teachers or both.

Engineering education in Sweden in the early 19th century was channelled through a number of schools, most notably the specialized School of Mining in Falun beginning in 1822, the Technological Institute in Stockholm in 1827, and the Chalmers Institute established two years later in the commercial city of Gothenburg on the Swedish west coast. The formation of these institutions had been preceded by debates on agricultural education in the Diet of the Estates, where the traditional agricultural methods of farmers stood against a more informed scientific way of pursuing farming especially connected to wealthier landowners (Torstendahl 1975: 44-55; Schaffer 1997). In the proposals for agricultural education, practice and theoretically informed knowledge were promoted in order for the teaching to rest on both scientific experiments and theories of agricultural chemistry. In political discussions about the most important type of knowledge for the improvement of agricultural practices, explicit references were made to foreign developments, especially those in Germany (Harwood 2005: 77-80). And eventually, after a number of parliamentary efforts stranded on the issue of costs, a private school was founded in 1834.

The same types of issues are recognized in the debates on higher technical education held in the Swedish Diet during the 1820s. The key argument for the introduction of publicly financed higher engineering training was that knowledge and reason would raise productivity in industry as well as in agriculture (Henriques 1917: 70–94; Torstendahl 1975: 56-58). Originally, the idea was to teach science to younger people already employed in workshops and elsewhere in order to tie physics and chemistry in particular to practical working life. Education, it was argued, should focus on scientific knowledge that could be used directly by those employed in production in order to raise productivity, which shows that the users and uses of the knowledge disseminated were the centre of attention from the very beginning. Those in favour of a new educational institution pointed out that prominent Swedish scientists had moved abroad instead of having been engaged domestically to raise industrial productivity. Simultaneously, they stressed that technical knowledge could not be deduced from the sciences, but that it must rely instead on the scientific systematization of experiences from industry.

All these standpoints regarding useful science were opposed, however, by the first vice chancellor of the Technological Institute in Stockholm, Gustaf Magnus Schwartz. Instead, he organized education along the lines of practical experience. The sciences were given a minor role, which soon led to bitter disputes between Schwartz, the board, and the Diet, where the proponents of scientific training raised their voices. These discussions make it clear that the curricula at institutions for higher engineering education were formed by both scientific and political debates. In both arenas, ideological considerations regarding the value of science in engineering rather than empirical proof marked the conclusions of the participants. Eventually, Schwartz had to resign as vice chancellor in 1845, opening the door for the introduction of scientific theory and experimental activities in the institute’s curriculum. At the Chalmers Institute in Gothenburg, the situation was somewhat different (Torstendahl 1975: 76-82). Here, the first vice chancellor stressed the importance of bringing the sciences into the curriculum already from the opening of the institute, and his appeal was soon put into practice.

### *The Academic Drift of Higher Vocational Education*

Higher engineering education was the subject of rather intense debate in many European countries during the second half of the 19th century (Manegold 1970; Lundgreen 1990; Fox and Guagnini 1993; Runeby 1976; Runeby 1978). In the 20th century, the signs of academic drift in Swedish higher engineering education became more visible, for instance, through an increase of public resources earmarked for experimental research in laboratories located in the engineering schools (Björck 2004: 287-295). In 1927, an engineering doctorate was introduced after much debate, and five years later, a programme in engineering physics was founded at the Technological Institute (Björck 1997). Both these novelties were conscious imports from Germany where they had been introduced some decades earlier. Another sign of academic drift in Swedish engineering schools depending on imports from abroad was the greater importance given to scientific credentials when appointing professors at the expense of industrial experience (Sundin 1981: 80-85; Larsson 1997: 88-101 and 191-212). Thus, despite the fact that the curricula were dominated by scientific and engineering topics, roughly balancing each other from the late 19th century onwards, there were other indications of the drift towards an increased valuation and assimilation of academic practices (Lindqvist 1993). It is important to stress that the arguments used to defend academic drift in Swedish engineering schools never relied on empirical support for the advantage of engaging scientifically trained teachers or expanding scientific subjects in the curriculum. Instead, proponents of both science- and practice-oriented teaching relied on assumptions as well as developments abroad to support their case.

The same type of academic drift has been visible in other institutions of higher vocational education—for example, schools devoted to medical and veterinary training—where new authorities in the form of professional societies and organizations influenced developments in much the same way (Gispen 1989; Lundgreen 1990). There were also other types of hybrid organizations where the management and production of knowledge gravitated in the 19th century. Best known perhaps are museums, which were built in many countries as a way to create and support national identity: including the collection, maintenance, and display of material; the dissemination of information to the public through exhibitions, tours, and educational activities; and research performed in relation to the collections (Hooper-Greenhill 1992; Knell 2007). Sweden was again no exception, and the best-known example of a Swedish museum functioning as a hybrid organization was the Swedish Museum of Natural History formed in 1831 (Lindman 1916; Broberg 1989; Beckman 1999; Beckman 2004). Like the botanical gardens in the 18th century, the museum relied on collections gathered and exhibited by the Royal Swedish Academy of Sciences.

## Industrial Research Institutes of the 20th Century

While institutions of higher vocational education were being exposed to academic drift in 20th-century Sweden, new types of hybrid organizations were contemplated. Again, the pattern had already been established abroad, where publicly and privately co-funded research institutes had been set up beginning in the second half of the 19th century. One important source of inspiration was the Kaiser-Wilhelm-Gesellschaft zur Förderung der Wissenschaften in Germany, which established institutes in different research areas of industrial interest such as chemistry (Johnson 1990). Similar organizations had also been introduced in Great Britain and America by the beginning of the 20th century. Modelled on the German Physikalisch-Technische Reichsanstalt founded in 1887 in Berlin, the British National Physics Laboratory and the National Bureau of Standards in America, founded in 1900 and 1901 respectively, dealt with materials testing as well as standardization issues and the control of scientific instruments (Cahan 1989; Moseley 1978; Pyatt 1983; Pyatt 1984). These efforts were intensified during World War I with the founding of the Department of Scientific and Industrial Research in Great Britain in 1916. The same year, the National Research Council was formed by the National Academy of Sciences in the US, largely funded by private foundations and with only loose connections to the federal government.

Like the earlier scientific academies and institutions of higher vocational education, Swedish industrial research institutes were formed under the influence of foreign prototypes. In Sweden, the new types of hybrid organizations set up to supply knowledge of interest to different industrial branches materialized primarily as industrial research institutes. An important precursor was the Materials Testing Laboratory (Materialprovningsanstalten), which had first been formed as a branch of the Swedish Steel Producers’ Association (Jernkontoret) to test metals and other materials to ensure quality and set standards. Towards the end of the 1890s, it was reorganized and put under the auspices of the Technological Institute in Stockholm, by this time renamed the Royal Institute of Technology.

### *Swedish Industrial Research Institutes as Hybrid Organizations*

The first regular industrial research institute in Sweden, however, was the Wood Pulp Research Association (Pappersmassekontoret), formed in 1917 by companies in the pulp business which contributed in proportion to their respective production. The owners thus commissioned the research (Sundin 1981: 19; Björck 2004: 221-5). The ongoing World War I was an important factor in its creation, but the economic crisis following the War in the early 1920s together with a lack of serviceable results put an end to the association in 1922. That year, the Swedish Institute for Metals Research (Metallografiska institutet) was inaugurated as the result of a collection held by Stockholm University College (Stockholms högskola) and the Swedish Steel Producers’ Association. The state participated as well by supplying housing and an annual allocation of money for the running of these institutes. The boom in international steel production, which had increased nearly a hundredfold between 1870 and 1910, as well as the expansion of domestic Swedish steel production, paved the way for the foundation of the Institute (Sundin 1981: 163-85; Sundin 1992). After only a few years, however, accountants complained that much of the turnover came from gifts rather than from contributions from the steel industry, thereby implying that the research at the Institute lacked relevance for the financiers. In the early 1930s, the Institute was reorganized after an initiative taken by the board, and in 1935 the director left his position after complaints from the board that too much of the Institute’s research was focused on basic research rather than the running of steel works.

It was no coincidence that these two research institutes, for wood pulp and metal respectively, represented the two most important branches of Swedish industry, at least when ranked according to export value. These branches carried the weight needed to create epistemic drift strong enough to lead to the foundation of formal organizations. But it was the planning of the third institute during World War I that caused a more long-lasting change in the institutional landscape of conveying publicly funded knowledge of industrial relevance. This was to be an institute for power and fuel research, whose formation was motivated not by the perceived status of energy as a profitable industry in its own right, but as a response to the problem of finding domestic supplies to meet the energy needs of Swedish industry in general. This problem was addressed by a number of representatives of government and industry, resulting in several public investigations and reports regarding such an institute. The outcome was the Royal Swedish Academy of Engineering Sciences (Kungliga Ingenjörsvetenskapsakademien), an organization housing several smaller institutes for consultative research and commissions (Sundin 1981; Peterson 1990; Brissman 2008).

During the interwar period, the Royal Swedish Academy of Engineering Sciences received substantial public as well as private funding for research on energy and building technologies. The financial backbone was the annual public contribution of between SEK 100,000 and 200,000 (today approximately corresponding to EUR 192,000 and EUR 384,000) for fuel and power research, a handsome sum considering that the total public allocation to the Royal Institute of Technology was SEK 360,000 (EUR 690,000) in 1919 (Sundin 1981: 98-106).^12^ Fuel and power research was partly established through the formation of no less than three different research institutes in the 1920s and early 30s, one for electrical heating, one for coal, and one for steam heating, all co-financed by public funding and private industry (Liander 1970; Stålhane 1970; Stenberg 1970; Cederquist 1970; cf. Härlin 1944). In 1929, the Concrete Laboratory (Cementlaboratoriet) was formed, and throughout the 1930s additional committees and commissions (e.g. for welding and corrosion research) were set up to assist technical areas in need of support (Giertz-Hedström 1970). In addition, the Academy of Engineering Sciences spent SEK 230,000 (EUR 441,000) funding approximately one hundred different studies during the 1920s, a sum that increased gradually so that about SEK 100,000 (EUR 192,000) was paid out annually towards the end of the 1930s (Ljungberg 1986, 36). These sums were, however, far from the SEK 400,000 (EUR 767,000) that the Academy had hoped to distribute annually, and lack of financial resources was a constant problem for the Academy throughout the interwar period (Sundin 1981: 128).

The standard toolkit for establishing research institutes early on included joint financial contributions from the industrial and public sectors, that is, private capital as well as tax revenue in one form or another. Therefore, the successful establishment of research institutes often relied on intense networking on the part of both academics and industrialists, the most important single organization promoting the formation of research institutes in the interwar period being the Academy of Engineering Sciences. The hybridity of early industrial research institutes in Sweden was mirrored in the fact that their first directors all had backgrounds in the Materials Testing Laboratory, where their interest in technical as well as scientific problems had been formed (Sundin 1981: 204-6). They all embraced what Eda Kranakis (1990) has called ‘hybrid careers’. As a result, the traditional academic view of science had to be accepted side by side with an ethos of utility. The industrial research institutes became a third arena, in addition to the earlier formed scientific academies and institutions of higher vocational education, where representatives of these two ideals of knowledge production could meet (Holmberg 2005; Holmberg 2010).

But interaction between the spheres of academia and industry could also lead to failed efforts. One area in which the Academy of Engineering Sciences attempted rather unsuccessfully to establish research was that of rationalization, especially as it related to the organization of industrial work processes and the analysis of working conditions in order to improve efficiency. Between the founding of the Academy in 1919 and the mid-1920s, efforts were made to establish a psycho-technical institute. It proved difficult to secure sufficient funding, however, and the initiative was eventually abandoned, mainly due to a lack of interest from the industrial sector (De Geer 1978: 117-58). Thus, irrespective of the tendency for epistemic drift prevailing among psychologists and other academics, the response of industry was too weak to result in a research institute in this case. Instead, the involved companies seem to have been satisfied with the existing methods of rationalization imported from abroad.

In one way, the failure to create a psycho-technical institute in the 1920s was an exception. Prior to 1919, when the Academy of Engineering Sciences appeared as an important hybrid organization, institutes and associations had been established exclusively in areas where revenue was large enough to support the type of uncertain, long-term investment that research often entailed. The Academy, however, seems to have established greater possibilities for funding the analysis of technical problems of a broader social interest.

In short, the first half of the 20th century saw an intensification in the creation of industrial research institutes financed jointly by government and private interests and coordinated by the Royal Swedish Academy of Engineering Sciences (Weinberger 1997: 42-5). In most cases, institutes and laboratories were formed to serve branches of industry where there was no distinguishable government agency acting as a major customer, typically branches related to natural resources such as pulp and ore. Regarding other technical problems of a broader scope, the establishment of the Academy of Engineering Sciences increased the possibilities of research funding, at least on a smaller scale.

It should be clear, then, that industrial research institutes qualify well as hybrid organizations, given their reliance on combinations of social practices drawn from the worlds of science and politics. It should be equally clear that the intended beneficiaries of these organizations were primarily industrial enterprises as well as branch organizations and their supporters, including unions and political parties. These beneficiaries influenced research problems as well as their solutions, and in doing so initiated and strengthened the process of epistemic drift. In university departments, however, the situation could differ. Historian of science Sven Widmalm (2004, 2008), for example, has shown how university-based research groups led by well-known Swedish Nobel laureates such as the chemists Theodor Svedberg and Arne Tiselius managed to balance funding from industry with academic freedom when studied over shorter time spans (a few decades rather than a century or more). His analyses of the network-building activities of Nobel laureates demonstrate that industrial funding did not necessarily hinder the free selection of research topics, and thus did not imply an unconditional epistemic drift.

### *The Academic Drift of Swedish Industrial Research Institutes*

Moreover, when compared over longer time spans, it is obvious that industrial research institutes focused more on research activities than on the dissemination of knowledge through meetings, teaching, and publications in the vernacular, as had been the focus of the scientific academies and institutions for higher vocational education. It is likewise apparent that this knowledge had to be both useful and accessible for the industrial branches with a financial interest in the institutes. Otherwise, the institutes could be dissolved or at least reorganized, as the examples of the institutes for wood pulp and metals research demonstrate.

Industrial research institutes did, however, exhibit a tendency for academic drift, the Swedish Institute for Metals Research serving as one early case in point. During and after World War II, the history of industrial research institutes became more tightly interwoven with the development of higher engineering education, leading to a substantial expansion of research resources for these institutes. The background was a public investigation into the possibility of establishing a national research policy in which the Swedish state would shoulder more financial and administrative responsibility for research activities beneficial to trade and industry (Nybom 1997: 45-52). When reviewing the different organizational alternatives in the early 1940s, the establishment of a national industrial research institute was highlighted as one of a number of feasible possibilities. The idea was abandoned, however, owing to the argument that the notion of a research institute separate from the education sector was outmoded, and that the existing institutions for higher engineering education should instead become more research intensive. The result was the introduction of research councils assigned to finance research in different areas by approving project applications from institutions for higher vocational education, universities, and other interested parties.

## Conclusions

By comparing the creation and developments of three types of hybrid organizations founded in the 18th, 19th, and 20th centuries, it has been the purpose of this article to answer the question of why new hybrid organizations are continuously formed. A few clues to the solution of this problem can now be formulated. Firstly, the foundation of new hybrid organizations seems to be the result of epistemic drift, that is, the valuation of research problems and results according to their relevance to politically and administratively determined goals, goals often created with different categories of knowledge users in mind, or through the initiative of the potential users themselves. When, for instance, different scientific academies were formed in the 16th and 17th centuries in Sweden and elsewhere, it was a way to put the natural sciences to use for national economical interests along utilitarian lines of thought, something many thought that the conservative universities were unsuccessful in doing. Also the establishment of higher vocational education in the 19th century must be seen as the result of epistemic drift. The best indication of this is that institutions for higher vocational training in Sweden and elsewhere were exclusively set up in areas where there was support external to the traditional sciences, for instance in engineering, physical education, medicine, dentistry, veterinary medicine, pharmaceutics, agriculture and forestry. Nevertheless, these new institutions were to a varying degree populated by academics from the universities and thus promoters of epistemic drift.

Secondly, hybrid organizations—at least those with a position in a status hierarchy, a geographic location, and an inclination to conform to prevailing perceptions of the relation between practice and theory according to Harwood’s model of institutional dynamics—tend to be exposed to academic drift so that the individuals involved and the value systems they embrace become increasingly similar to those found in universities. In all examples recounted here, however, this process has been very slow, not detectable until after several decades or even centuries. In addition, hybrid organizations are not determined to drift academically. Instead, this process has to be documented in each separate case.

Thirdly, since older hybrid organizations often prevail as new ones are introduced, they form historical layers like superposed sediments. From an international perspective, the most obvious indication of this is, of course, that there are very few cases of hybrid organizations of the types discussed here to have been abolished once they have acquired some measure of recognition (Hallonsten and Heinze 2012). Sure enough, such organizations do exist, but they have generally been short-lived experiments that never got off the ground rather than long-lasting organizations with potential to drift.

To reach these conclusions, I have accounted for the relevant generalizable international developments—the wave of scientific academies formed in Europe in the 17th and 18th centuries, the equally distinct trend of institutions of higher vocational education in the 19th century and the somewhat less marked movement of industrial research institutes of the 20th century—and the different historical non-generalizable processes these international tendencies led to in Sweden. Each case of hybrid organizations thus demonstrates the same type of causal chain where generalizable international developments led to non-generalizable national processes showing the nature of epistemic and academic drift in Swedish hybrid organizations.

Noting, however, that Sweden consistently strove to implement existing international policy trends during the periods in question, not the least marked by the recurring international influences on the historical processes reviewed here, I claim that the observations made regarding the dynamics of epistemic and academic drift in Sweden are generalizable and can be applied to other similar contexts where ambitions to follow international trends dominate together with a willingness to let these trends influence local and national processes resulting in policy convergence. This claim is also valid when taking into account some Swedish peculiarities in research policy resulting from, among other things, the Nobel Foundation and its prizes established by the turn of the 20th century, which led to the formation of research institutes during the first half of the 20th century, and strengthened the connections between labour and capital (Crawford 1984; Friedman 2001; Gribbe, Lundin, and Stenlås 2010).

Of course, exceptions from these general observations come to light when developments in different countries are compared in detail. For instance, the American institutional system of knowledge dissemination in the agricultural sciences was built up so that educational institutions appeared before research facilities, which in turn appeared before information dissemination through academic journals and other forms of printed media (Cash 2001). In Sweden, the same sector demonstrates a different institutionalization process where journals and congregations for dissemination established in the 18th century were followed by organizations for training in the first half of the 18th century and research establishments later on in the same century (Edling 2003). It is needless to point out that such differences are important, and any attempt to explain why new hybrid organizations are formed is bound to rely on generalizations. Here, one such generalization is that epistemic drift characterizes the foundation of different types of hybrid organizations; another is that academic drift characterizes the historical development of some of them, and occasionally ensures their survival after their initial purpose has been abandoned or forgotten.

With all these reservations in mind, an answer to the problem of the formation of new hybrid organizations can be proposed. The process of academic drift has often entailed a gradual marginalization of the knowledge users whom the different historical hybrid organizations had originally been formed to serve. Only seldom have hybrid organizations sought to make themselves relevant to new categories of knowledge users as the original ones have been marginalized. Instead, they have tended to accede to ideals supported by traditional academic organizations with higher status in terms of knowledge management, primarily universities. Through this process, in which older hybrid organizations tend to gradually turn their focus away from the original users, demand has been generated for the founding of new hybrid organizations.

Note that this answer points to organizational dynamics rather than changing historical conjunctures in the uses of science. Simultaneously, the hybrid organizations analyzed here have not been founded with indifference to the organizational ideals dominating their respective time of foundation. Instead, they have all responded to differing notions of the most efficient way to make knowledge relevant: in the 18th century, heterogeneous congregations supported by members of networks stretching from universities over commerce and into politics; in the 19th century, vocational education supported by professional organizations and the state; and in the 20th century, industrial research institutes focusing on knowledge production supported by scientists and engineers pursuing hybrid careers.

## Footnotes

1 Compare to hybrid forums as defined by Callon, Lascoumes and Barthe (2009: 18). There are also more specifically defined types of organizations for exchange between scientific expertise and policymakers (Braun 1993; Guston 2000; van der Meulen 2003). On the differences between boundary organizations and intermediary agencies, see Guston (2001). Regarding weaknesses of principal-agent theory, see Morris (2003) and Shove (2003).

2 For a similar analysis regarding post-war Swedish science policies, see Edqvist (2003).

3 Here, the word “commercially” has been added to the original definition as given in Elzinga (1984). See also Elzinga (1997).

4 Regarding users, see Shove and Rip (2000) and Davenport, Leitch, and Rip (2003).

5 For a definition and thorough review, see Harwood (2010).

6 Not ‘Economic Scientific Academy for the Promotion of the Useful Sciences’ as proposed in McClellan III (1985: 34), see Hildebrand (1939a, b: 266-272), Koerner (1994, 1999), Lindroth (1967: 217), and Uggla (1940).

7 The sentence quoted reads in its entirety: ‘All those sciences and arts possessing real utility for the commonwealth are to be subject of attentive and devout efforts of the Academy.’ In original Swedish: ‘Alla de wetenskaper och konster som äga någon wärkelig nytta i det allmänna wäsendet, blifwa ämnen till Academiens uppmärksamma och öma bemödanden’, see Hildebrand (1939a, b: 760).

8 ‘ett blandat sällskap’. Quotation from Lindroth I (1967: 27).

9 ‘beskyddare och beskyddade’. Quotation from Lindroth II (1967: 90).

10 ‘för de på kemiska och fyiska grunder beroende näringarna’. Quotation from Henriques (1917: 82). See also Lindroth, II (1967: 283-291).

11 Regarding botanical gardens, see Spary (2000).

12 The conversion of historical currency into present Euros relies on consumer price indices in Edvinsson and Söderberg (2010), Table A8.1, as well as the exchange rate between SEK and EUR averaging 8,87 in January 2012.
